# The Australian longitudinal study on male health sampling design and survey weighting: implications for analysis and interpretation of clustered data

**DOI:** 10.1186/s12889-016-3699-0

**Published:** 2016-10-31

**Authors:** Matthew J. Spittal, John B. Carlin, Dianne Currier, Marnie Downes, Dallas R. English, Ian Gordon, Jane Pirkis, Lyle Gurrin

**Affiliations:** 1 Centre for Mental Health, Melbourne School of Population and Global Health, The University of Melbourne, Melbourne, VIC 3010 Australia; 2 Centre for Epidemiology and Biostatistics, Melbourne School of Population and Global Health, The University of Melbourne, Melbourne, 3010 Australia; 3 Murdoch Childrens Research Institute, Royal Children’s Hospital, Parkville, 3052 Australia; 4 Department of Paediatrics, Melbourne Medical School, The University of Melbourne, Melbourne, 3010 Australia; 5 Statistical Consulting Centre, School of Mathematics and Statistics, The University of Melbourne, Melbourne, 3010 Australia

## Abstract

**Background:**

The Australian Longitudinal Study on Male Health (Ten to Men) used a complex sampling scheme to identify potential participants for the baseline survey. This raises important questions about when and how to adjust for the sampling design when analyzing data from the baseline survey.

**Methods:**

We describe the sampling scheme used in Ten to Men focusing on four important elements: stratification, multi-stage sampling, clustering and sample weights. We discuss how these elements fit together when using baseline data to estimate a population parameter (e.g., population mean or prevalence) or to estimate the association between an exposure and an outcome (e.g., an odds ratio). We illustrate this with examples using a continuous outcome (weight in kilograms) and a binary outcome (smoking status).

**Results:**

Estimates of a population mean or disease prevalence using Ten to Men baseline data are influenced by the extent to which the sampling design is addressed in an analysis. Estimates of mean weight and smoking prevalence are larger in unweighted analyses than weighted analyses (e.g., mean = 83.9 kg vs. 81.4 kg; prevalence = 18.0 % vs. 16.7 %, for unweighted and weighted analyses respectively) and the standard error of the mean is 1.03 times larger in an analysis that acknowledges the hierarchical (clustered) structure of the data compared with one that does not. For smoking prevalence, the corresponding standard error is 1.07 times larger. Measures of association (mean group differences, odds ratios) are generally similar in unweighted or weighted analyses and whether or not adjustment is made for clustering.

**Conclusions:**

The extent to which the Ten to Men sampling design is accounted for in any analysis of the baseline data will depend on the research question. When the goals of the analysis are to estimate the prevalence of a disease or risk factor in the population or the magnitude of a population-level exposure-outcome association, our advice is to adopt an analysis that respects the sampling design.

## Background

Like many large-scale health surveys, the Australian Longitudinal Study on Male Health (Ten to Men) used a complex sampling scheme. This choice was made because sampling the target population using a simple random sample was not feasible. Sampling theory therefore plays an important role in our study design because it provides a framework for efficiency gains [1]. In Ten to Men, the key elements of the sample design were the use of stratification, multi-stage sampling and cluster sampling to select prospective participants and invite them to take part in the study. This design has implications for the analysis of data from Ten to Men for both inferences about population means or prevalences, and for quantifying the magnitude of associations between exposures and outcomes. Such analysis implications are, however, often poorly understood. At the extreme, views differ on whether to *always* adjust for aspects of the study design and sampling scheme at the analysis stage (including accounting for unequal sampling fractions using inverse-probability-of-selection sampling weights) or to *never* adjust. Korn and Graubard [2] give an excellent example of this controversy using US National Health and Nutrition Examination Surveys (NHANES). At the heart of this debate is a trade-off between mitigating against bias in estimation while faithfully representing the repeated sampling variation of the corresponding estimators in order to ensure accurate inferences.

Our aims here are to (1) describe each of these competing elements as they relate to Ten to Men; (2) detail and discuss the calculation of inverse-probability-of-selection sampling weights; and (3) provide recommendations for analyses that acknowledge these aspects of the design. We use a continuous variable (weight in kilograms) and a binary variable (current smoking status: smoker or non-smoker) as illustrations throughout. Our attention is restricted to the baseline (i.e. prevalent) wave of data collection. Our analyses are conducted in Stata [3], but the same principles and procedures apply to other statistical packages.

## Methods

### Overview of the ten to men sampling design

#### Stratification

When stratification is used in a survey design, it refers to the population being partitioned into groups prior to selection of the sample [4]. Samples are then taken independently within each stratum.

The Australian Statistical Geographic Standard [5] (ASGS) used by the Australian Bureau of Statistics (ABS) classifies each location within Australia as belonging to one of five levels of remoteness: “Major Cities”, “Inner Regional”, “Outer Regional”, “Remote” and “Very Remote”. It was not feasible to survey remote and very remote regions because of the travel time required for fieldworkers to recruit potential participants into the study (less than 2.3 % of the population lives in rural and remote Australia, an area that covers most of the country), so the study was restricted to sampling from the first three strata, that is, the major cities, inner regional and outer regional areas. Inner and outer regional areas were over-sampled to ensure that questions related to regional disparities in male health could be addressed adequately. These areas therefore represented 23 % and 20 % of the sample at baseline (for inner and outer regional area, respectively) compared with population proportions of 18 % and 9 %.

### Multi-stage sampling

Sitting alongside the ASGS classification of remoteness is the ABS division of the population into “statistical areas”. The smallest units are Mesh Blocks (there are about 350,000 of these, containing on average about 75 people each), which aggregate into Statistical Area 1s (SA1s, with an average of 400 people and ranging from 200 to 800), then SA2s (averaging 10,000 people with a range of 3,000–25,000), SA3s, SA4s and finally SA5s which are the six Australian States and two Territories.

Ten to Men employed a multi-stage design. For the major cities stratum, SA1s were sampled first (proportional to size, where size referred to the number of boys according to the ABS 2011 Census of Population and Housing) and all households were sampled within SA1s. For the inner and outer regional strata, SA2s were randomly sampled first (also proportional to size using the same definition as that used for major cities) and then a fixed number of SA1s were randomly sampled within SA2s; at the final stage, households were sampled within SA1s. This additional step in the hierarchy of sampling SA2s for the inner and outer regional strata was introduced to reduce the distance fieldworkers had to travel.

### Clustering

For all three strata, all eligible males within an eligible household were invited to participate in the study (see Table 1 in *Australian Longitudinal Study on Male Health – Methods* in this collection for a definition of the eligibility criteria). Thus, within a stratum, Ten to Men can be described as a cluster sample of eligible households, with SA1s defining the cluster. Households were therefore an additional level in the completely-nested hierarchy implied by the multi-stage sampling design.

**Table 1:** Estimated mean weight (kg) and prevalence of smoking using seven different approaches

	Adjustment for clustering?	Levels of sampling hierarchy used?	Adjustment for stratification?	Sample weights used?	Weight, kg Mean (95 % CI)	Current smoker Prevalence (95 % CI)
A	No	None	No	No	83.9 (83.6 to 84.2)	18.0 (17.4 to 18.6)
B	No	None	No	Yes	81.4 (80.9 to 81.9)	16.6 (15.9 to 17.4)
C	Yes	SA1, SA2, household	No	No	84.0 (83.5 to 84.5)	NR
D	Yes	SA1	Yes	Yes	81.4 (80.8 to 81.9)	16.7 (15.7 to 17.6)
E	Yes	SA1	No	Yes	81.4 (80.8 to 81.9)	16.7 (15.7 to 17.6)
F	Yes	SA2	Yes	Yes	81.4 (80.8 to 81.9)	16.7 (15.6 to 17.7)
G	Yes	SA2	No	Yes	81.4 (80.8 to 81.9)	16.7 (15.6 to 17.7)

### Sample weights

The sampling design of Ten to Men implies that individuals within the major cities stratum did not have equal probabilities of selection because individuals living in SA1s with a larger number of boys (according to the ABS 2011 Census of Population and Housing) are more likely to be invited to participate since sampling was proportional to size where size refers to the number of boys. Although individuals in the inner and outer regional strata did, in theory, have equal probabilities of selection (due to the selection of the fixed number of SA1s within each SA2 effectively “cancelling out” the sampling of SA2s with probability proportional to their size), this was violated in practice due to variation in the participation fractions between households, SA1s and SA2s. This variability was an issue for the major cities stratum as well.

Sampling weights can be used to address bias in estimation due to unequal sampling fractions and to account for non-response when estimating a population parameter. These sampling weights are calculated as the inverse of the individual probability of participation. For inner and outer regional participants the weights are the inverse of the product of (1) the probability of an SA2 being selected: (2) the probability of an SA1 within SA2 being selected; and (3) the probability of an individual within an SA1 both agreeing to participate and providing usable data. For major city participants, the weights are the inverse of the product of (1) the probability of an SA1 being selected and (2) the probability of an individual with an SA1 agreeing to participate and provide usable data. Where a stratum is under-represented in the sample compared to the population then the sampling weights will up-weight data from individuals in that stratum in the analysis. Details on the calculations of the baseline sampling weights for Ten to Men are given in Appendix 1.

## Results and discussion

### Implications for estimating population means, prevalences and totals

Estimating means, prevalences or totals from a complex survey as though they were generated from a simple random sample has the potential to generate biased estimates and for the stated precision of these estimates to differ from the variability that we would observe in them under repeated sampling. The multi-stage sampling and selection of household clusters must therefore be acknowledged and accommodated when estimating population parameters. This can be done by either specifying a full multi-level model (by using a generalised linear mixed model) or by using a set of “survey” commands, both of which are available in most standard statistical software packages (including Stata). The multi-level model approach allows us to account for all levels of the hierarchy (i.e., individuals nested within households, households nested within SA1s etc.) but does not allow the effect of the sample weights to be incorporated into the analysis at the level of the individual participant (only group level weights are allowed at least for the suite of mixed models procedures we considered in Stata, e.g., mixed, melogit, and meglm). This means the estimates generated from this procedure may be biased.

The survey commands (at least those implemented by Stata and other major programs) only allow proper accounting for clustering at the top level of the multi-stage sampling hierarchy. This distinction is relevant in Ten to Men because for major cities, SA1s sit at the top of the hierarchy, whereas for the inner and outer regional strata, the larger SA2s were the first unit to be sampled. However, these commands do allow sample weights to be specified in the analysis. Consequently there is not a single procedure implemented in the commonly used software platforms that can account for the multi-stage design of the survey (which affects the calculation of the variance estimates) and produce unbiased estimates of population parameters when using data from all three strata (which the weights are intended to address).

We now demonstrate four approaches that reflect different ways of dealing with these issues when estimating a population parameter. Table 1 shows the mean weight (in kilograms) and a 95 % confidence interval (CI) for the corresponding population parameter calculated with no adjustment for the survey design or the sample weights (row A), no adjustment for the survey design but using sample weights (row B), using multi-level modelling without adjustment for the weights (row C) and using survey commands that allow for different combinations of adjustment for the multi-stage design and stratification as well as inclusion of the weights (rows D to G). Rows D and E present results using the SA1 as the primary sampling unit (PSU, the top level of the sampling hierarchy) whereas rows F and G uses the SA2 as the PSU. Rows D and F present estimates that are adjusted for stratification but E and G do not. Different estimates of smoking prevalence using the same analytic methods are also provided in Table 1, with the exception that the result from a multi-level logistic regression is excluded because such models do not estimate a parameter that has a population-level interpretation [6] and are thus not directly comparable to the other estimates.

As expected, the estimates of the population mean weight and the confidence intervals differ depending on the extent to which the sampling design characteristics are accommodated by the estimation procedure. When the population mean is estimated under the assumption of simple random sampling (row A), the mean weight is 83.9 kg (95 % CI 83.6 to 84.2 kg). Repeating this analysis but incorporating adjustment for the sample weights (row B) gives a mean weight of 81.4 (95 % CI 80.9 to 81.9). Using a multi-level modelling strategy that adjusts for the correlated observations within households, SA2s and SA1s (but does not adjust for stratification or sample weights) gives a similar mean to the unweighted analysis of 84.0 kg (95 % CI 83.5 to 84.5 kg) (row C). This mean appears to be biased probably because it does not account for the sample weights. Population estimates that account for the top level of the sampling hierarchy as well as weighting (and either with or without adjustment for stratification) are all identical to the level of precision reported (rows D to G). For example, when the SA1 is used as the PSU and the estimates are adjusted for stratification, the mean is 81.4 kg (95 % CI 80.8 to 81.9). This estimate is the same for all other combinations of PSU (SA1 vs SA2) and adjustment for stratification (no adjustment vs adjustment).

The results for analysing a binary variable, current smoking status, paint a similar picture. The estimate of the population prevalence is highest when no adjustment is made for the sampling scheme. It is lower when adjustments are made for this, with no appreciable difference between using the SA1 or the SA2 as the PSU or making adjustment for stratification.

### Implications for estimating associations

Estimates of the association between variables (e.g., self-rated health and weight or smoking status) may also be affected by how the sampling scheme is treated in the analysis [7, 8]. Most modern statistical programs have commands that enable linear, logistic and other multivariable regression techniques to be used that account for stratification, multi-stage sampling and sample weights. The question that arises is: When should these commands be used? The conditions under which clustering can be ignored in the analysis of data are quite restrictive. In general, to be able to ignore clustering without producing variance estimates (and therefore confidence intervals) that are too narrow, we require at least that the distribution of the outcome of interest within given levels of risk factors and covariates does not differ between clusters [2]. In most scenarios it is far from obvious that this condition is satisfied: It is difficult to test empirically and will be untestable for unmeasured risk factors, covariates and confounders. Moreover, introducing adjustments for a stratified, multi-stage, clustered sampling scheme and for sample weights to accommodate unequal sampling fractions can lead to estimates that are highly variable [4]. This has implications for detecting associations between the exposure and an outcome. Against this, theoretical work by Scott and Holt [7] and by Neuhaus and Segal [8] show that estimates of measures of association in linear and logistic regression models are generally unbiased if we fail to account for clustering in the analysis. Lumley [9] makes a case for not using sample weights based on the argument that regression models often includes confounders and covariates that explain the variation in weights. This adjusts for any distortions in estimating the magnitude of the association between the exposure and the outcome that would have resulted from ignoring unequal sampling fractions. It is true that for some population-level measures of association the unbiasedness and variability of their estimates will not depend on whether or not the analysis incorporates the stratum-specific sampling fractions, but a full description of such scenarios is beyond the scope of this paper (see Lumley [9]). It is worth noting, however, that the variance of the measures of association, and as a consequence, the standard errors, confidence intervals and p-values calculated from them may be incorrect. Scott and Holt noted the extent of this mis-specified precision is generally less severe than when estimating means and prevalences.

We explore these issues with data examining the association between self-rated health and weight using linear regression. The measure of interest is the difference in mean weight (in kilograms) between two groups: those reporting excellent or very good health and those reporting good, fair or poor health. We compare results under a variety of conditions: where there is adjustment for the multi-stage design (no adjustment, adjustment for all stages of the hierarchy using multi-level modelling, adjustment using the SA1 as the PSU, adjustment using the SA2 as the PSU), adjustment for stratification (no adjustment, adjustment using the stratification variable as a covariate, adjustment using the survey command), and use of sample weights (yes or no). We also examine the association between self-rated health and smoking status using logistic regression, where the effect size of interest is an odds ratio. We again omit the results from analyses that use a multi-level logistic model for the same reasons discussed in the previous section.

In an analysis that makes no adjustment for the multi-stage design or for stratification or weighting (Table 2, row A), the mean difference between the two groups is −5.1 kg (95 % CI −5.8 to −4.5 kg). That is, those who describe themselves as having very good or excellent health report are, on average, 5.1 kg lighter than those who have good, fair or poor health. Adjusting for stratification by using a series of indicator variables for remoteness to enter it into the model as a categorical variable (row B) also gives a mean difference of −5.1 kg with 95 % CI −5.7 to −4.4 kg. Repeating the analysis in row A but with the use of sample weights to adjust for bias gives a smaller difference of −4.4 kg, but with a wider confidence interval than observed previously (95 % CI −5.6 to −3.3). Adjustment for stratification makes only a small difference to this result (row D).

**Table 2:** Estimated difference in mean weight and estimated odds ratios between participants with excellent or very good health and participants with good, fair or poor health

	Adjustment for clustering?	Levels of sampling hierarchy used?	Adjustment for stratification?	Sample weights used?	Weight: Excellent or very good health (vs good, fair or poor health): Difference (95 % CI)	Current smoker: Excellent or very good health (vs good, fair or poor health): Odds Ratio (95 % CI)
A	No	None	No	No	−5.1 (−5.8 to −4.5)	0.39 (0.36 to 0.42)
B	No	None	Yes	No	−5.1 (−5.7 to −4.4)	0.39 (0.36 to 0.43)
C	No	None	No	Yes	−4.4 (−5.6 to −3.3)	0.42 (0.37 to 0.47)
D	No	None	Yes	Yes	−4.3 (−5.5 to −3.2)	0.42 (0.38 to 0.47)
E	Yes	SA1, SA2, household	No	No	−4.9 (−5.5 to −4.2)	NR
F	Yes	SA1, SA2, household	Yes	No	−4.8 (−5.5 to −4.2)	NR
G	Yes	SA1	Yes	Yes	−4.4 (−5.5 to −3.2)	0.42 (0.37 to 0.47)
H	Yes	SA1	Yes	No	−5.1 (−5.8 to −4.4)	0.39 (0.35 to 0.42)
I	Yes	SA2	Yes	Yes	−4.4 (−5.5 to −3.3)	0.42 (0.37 to 0.47)
J	Yes	SA2	Yes	No	−5.1 (−5.9 to −4.4)	0.39 (0.35 to 0.43)

Repeating the analysis to account for all stages of sampling using a multilevel model (rows E and F) gives a mean difference of −4.9 kg (95 % CI −5.5 to −4.2), with further adjustment for stratification giving a difference of −4.8 kg (95 % CI −5.5 to −4.2). As with estimating population prevalences using multi-level models, it is not possible to easily account for the sample weighting in this context.

The final four rows in Table 2 show results obtained using the survey commands to estimate the population mean difference. When SA1s are defined as the PSU and sample weights are used (row G), the mean difference between the two groups is −4.4 kg (95 % CI −5.5 to −3.2). When no weights are used, the difference is −5.1 kg (95 % CI −5.8 to −4.4). Using the SA2 as the PSU gives similar results (rows I and J).

Thus, the estimate of the mean difference ranges from −4.3 kg to −5.1 kg. Taken as a whole, these results suggest that it is the adjustment for the sample weights that has the biggest impact on the results, with the adjustments for the sampling hierarchy and stratification having relatively minor influences on the estimate of the effect size. Nonetheless, all analyses would lead to the conclusion that the mean weight differs between the two groups, with those who have excellent or very good health being 4 to 5 kg lighter than those who have good, fair or poor health. This suggests that the way in which the sample design is accounted for makes some difference to the estimate of this measure of association on this occasion. This is supported by the second analysis in Table 2, which shows that the odds of being a current smoker are approximately 60 % lower for those with excellent or very good health compared with the odds for those with good, fair or poor health regardless of the way in which the study design is accommodated in the analysis.

### Conclusion

Analyses of baseline data from Ten to Men will require explicit adjustment (through the use of sampling weights or procedures for clustering) for the sampling design in order to generate unbiased estimates with reliable measures of their precision that reflect their variability under repeated sampling. The application of adjustments will depend largely on the particular research question and the proposed statistical analysis. While we have illustrated these concepts in the context of the Ten to Men study, the issues are relevant to all clustered survey designs.

For estimates of a population prevalence and totals, the sampling design (including sample weights) should be adjusted for, since the estimators will (most likely) be biased and its precision understated if unadjusted, because the sampling variability will depend on the sampling fractions and hierarchical structure of the data. The issues are defining the PSU (SA1 or SA2) and whether or not to adjust for stratification. Regarding the PSU, our results show little difference in practice between using the SA1 or the SA2 as the PSU. Our recommendation is therefore to treat SA1s as the PSU. Similarly, while adjustment for stratification made no appreciable difference in this instance, we also recommend adjusting for stratification. In support of this, Appendix 2 contains the variable names and the Stata code (using the svy suite of commands or its equivalent in other packages) that allow this recommendation to be implemented. It is less clear with regard to measures of association between exposure and outcome whether ignoring the sampling design and, in particular, not using weights in analyses, will lead to biased estimates. On balance, we favour an approach that respects the sampling design and therefore incorporates this information into the calculation of any effect sizes.

Some researchers may find it helpful to conduct sensitivity analyses, where they compare unadjusted and adjusted estimates of prevalence and associations to determine which of the results are sensitive to the extent that the sampling scheme is accommodated in the analysis. We support this, with the proviso that a statistical analysis plan be prepared prior to commencing analysis (see Thomas and Peterson [10] or Rubin [11] for excellent discussions on the value of doing this in observational studies).

## Appendix 1

### Calculating weights using inverse probability of selection

In this appendix we provide details of the calculation of the individual-level sampling weights, which are based on the inverse of the individual probability of selection for invitation to participate in the study.

Denote

- the number of SA1s selected across the remoteness stratum as K;
- the k^th^ SA1 as SA1_k_ for k = 1, 2, …, K;
- the true number of in-scope boys, young men and adults currently residing in SA1_k_ as T^B^_k_, T^YM^_k_ and T^A^_k_ respectively;
- the number of “ABS” boys, young men and adults in SA1_k_ according to the 2011 Australian Bureau of Statistics (ABS) Census of Population Housing as C^B^_k_, C^YM^_k_ and C^A^_k_ respectively;
- the number of boys, young men and adults who were “found” (approached and invited to participate in the study) in SA1_k_ be F^B^_k_, F^YM^_k_ and F^A^_k_ respectively; and
- the number of boys, young men and adults who provided “usable” data in SA1_k_ as U^B^_k_, U^YM^_k_ and U^A^_k_ respectively. Note that all participants who provided usable data were, by definition, found (see previous dot point), but they may or may not have been represented in the SA1_k_ in which they resided when recruited to “Ten to Men” during the 2011 Census.

***Major Cities Stratum***

***Boys***

For a boy in SA1_k_ the probability of (1) that SA1_k_ being selected; (2) that boy then being found; and finally (3) that boy providing usable data, denoted P^B^_k_, is

1 $$ \begin{array}{l}{{\mathrm{P}}^{\mathrm{B}}}_{\mathrm{k}} \propto \Pr \left(\mathrm{S}\mathrm{A}{1}_{\mathrm{k}}\mathrm{selected}\right) \times \Pr \left(\mathrm{Boy}\ \mathrm{found}\ \Big|\mathrm{S}\mathrm{A}{1}_{\mathrm{k}}\mathrm{selected}\right) \times \Pr \left(\mathrm{Boy}\ \mathrm{provides}\ \mathrm{usable}\ \mathrm{data}\ \Big|\ \mathrm{Boy}\ \mathrm{found}\right)\\ {}{{\mathrm{P}}^{\mathrm{B}}}_{\mathrm{k}}\kern1.25em \propto {{\mathrm{C}}^{\mathrm{B}}}_{\mathrm{k}}\times {{\mathrm{F}}^{\mathrm{B}}}_{\mathrm{k}}/{{\mathrm{T}}^{\mathrm{B}}}_{\mathrm{k}}\times {{\mathrm{U}}^{\mathrm{B}}}_{\mathrm{k}}/{{\mathrm{F}}^{\mathrm{B}}}_{\mathrm{k}}\\ {}\propto {{\mathrm{C}}^{\mathrm{B}}}_{\mathrm{k}}\times {{\mathrm{U}}^{\mathrm{B}}}_{\mathrm{k}}/{{\mathrm{T}}^{\mathrm{B}}}_{\mathrm{k}}\end{array} $$

Note that the number of found boys F^B^_k_ cancels out of calculation of P^B^_k_. We calculate the weight W^B^_k_ as inversely proportional to P^B^_k_

$$ \begin{array}{l}{{\mathrm{W}}^{\mathrm{B}}}_{\mathrm{k}}\kern1em \propto 1/{{\mathrm{C}}^{\mathrm{B}}}_{\mathrm{k}}\times {{\mathrm{T}}^{\mathrm{B}}}_{\mathrm{k}}/{{\mathrm{U}}^{\mathrm{B}}}_{\mathrm{k}}\\ {}\propto {{\mathrm{T}}^{\mathrm{B}}}_{\mathrm{k}}/{{\mathrm{C}}^{\mathrm{B}}}_{\mathrm{k}}\times 1/{{\mathrm{U}}^{\mathrm{B}}}_{\mathrm{k}}\end{array} $$

where the constant of proportionality is calculated so that the sum of the weights for all boys providing usable data across all selected SA1’s in the Major Cities stratum is N^B^. The true number of in-scope boys T^B^_k_ residing in SA1_k_ can never be known, so we must make an assumption about its value. We assume that T^B^_k_ = C^B^_k_. This assumes that ABS number of boys from the 2011 Australian Census is the true number of in-scope boys residing in SA1_k_. This assumption gives

$$ {{\mathrm{W}}^{\mathrm{B}}}_{\mathrm{k}}\propto 1/{{\mathrm{U}}^{\mathrm{B}}}_{\mathrm{k}} $$

Here the weight for SA1_k_ is inversely proportional to the number of boys providing usable data for SA1_k_, effectively up-weighting data from SA1s with low participation fractions and thus poor participation.

***Young Men and Adults***

Following the calculation in Equation (1) above we get

$$ \begin{array}{l}{{\mathrm{P}}^{\mathrm{YM}}}_{\mathrm{k}}\kern0.75em \propto {{\mathrm{C}}^{\mathrm{B}}}_{\mathrm{k}}\times {{\mathrm{F}}^{\mathrm{YM}}}_{\mathrm{k}}/{{\mathrm{T}}^{\mathrm{YM}}}_{\mathrm{k}}\times {{\mathrm{U}}^{\mathrm{YM}}}_{\mathrm{k}}/{{\mathrm{F}}^{\mathrm{YM}}}_{\mathrm{k}}\\ {}\propto {{\mathrm{C}}^{\mathrm{B}}}_{\mathrm{k}}\times {{\mathrm{U}}^{\mathrm{YM}}}_{\mathrm{k}}/{{\mathrm{T}}^{\mathrm{YM}}}_{\mathrm{k}}\end{array} $$

$$ \begin{array}{l}{{\mathrm{W}}^{\mathrm{YM}}}_{\mathrm{k}} \propto 1/{{\mathrm{C}}^{\mathrm{B}}}_{\mathrm{k}}\times {{\mathrm{T}}^{\mathrm{YM}}}_{\mathrm{k}}/{{\mathrm{U}}^{\mathrm{YM}}}_{\mathrm{k}}\\ {}\propto {{\mathrm{T}}^{\mathrm{YM}}}_{\mathrm{k}}/{{\mathrm{C}}^{\mathrm{B}}}_{\mathrm{k}}\times 1/{{\mathrm{U}}^{\mathrm{YM}}}_{\mathrm{k}}\end{array} $$

which, when we replace T^YM^_k_ with C^YM^_k_, becomes

$$ {{\mathrm{W}}^{\mathrm{YM}}}_{\mathrm{k}} \propto {{\mathrm{C}}^{\mathrm{YM}}}_{\mathrm{k}}/{{\mathrm{C}}^{\mathrm{B}}}_{\mathrm{k}}\times 1/{{\mathrm{U}}^{\mathrm{YM}}}_{\mathrm{k}} $$

Similarly for adults we get

$$ {{\mathrm{W}}^{\mathrm{A}}}_{\mathrm{k}}\kern1em \propto {{\mathrm{C}}^{\mathrm{A}}}_{\mathrm{k}}/{{\mathrm{C}}^{\mathrm{B}}}_{\mathrm{k}}\times 1/{{\mathrm{U}}^{\mathrm{A}}}_{\mathrm{k}} $$

***Inner and Outer Regional Strata***

***Boys***

For the inner and outer regional strata SA1s have equal probability of selection, so the term Pr(SA1_k_ selected) does not vary within a remoteness stratum and can therefore be absorbed in the constant of proportionality.

To illustrate, for a boy in SA1_k_ the probability of selection P^B^_k_ is

2 $$ \begin{array}{l}{{\mathrm{P}}^{\mathrm{B}}}_{\mathrm{k}\kern1.5em } \propto \Pr \left(\mathrm{Boy}\ \mathrm{found}\ \Big|\mathrm{S}\mathrm{A}{1}_{\mathrm{k}}\mathrm{selected}\right) \times \Pr \left(\mathrm{Boy}\ \mathrm{provides}\ \mathrm{usable}\ \mathrm{data}\ \Big|\ \mathrm{Boy}\ \mathrm{found}\right)\\ {}\propto {{\mathrm{F}}^{\mathrm{B}}}_{\mathrm{k}}/{{\mathrm{T}}^{\mathrm{B}}}_{\mathrm{k}}\times {{\mathrm{U}}^{\mathrm{B}}}_{\mathrm{k}}/{{\mathrm{F}}^{\mathrm{B}}}_{\mathrm{k}}\\ {}\propto {{\mathrm{U}}^{\mathrm{B}}}_{\mathrm{k}}/{{\mathrm{T}}^{\mathrm{B}}}_{\mathrm{k}}\end{array} $$

Replacing T^B^_k_ with C^B^_k_ based on the assumption above, gives

$$ {{\mathrm{P}}^{\mathrm{B}}}_{\mathrm{k}}\kern1.25em \propto {{\mathrm{U}}^{\mathrm{B}}}_{\mathrm{k}}/{{\mathrm{C}}^{\mathrm{B}}}_{\mathrm{k}} $$

and therefore

$$ {{\mathrm{W}}^{\mathrm{B}}}_{\mathrm{k}}\kern1em \propto {{\mathrm{C}}^{\mathrm{B}}}_{\mathrm{k}}/{{\mathrm{U}}^{\mathrm{B}}}_{\mathrm{k}} $$

***Young Men and Adults***

Similarly for young men and adults we get W^YM^_k_ ∝ C^YM^_k_/U^YM^_k_ and W^A^_k_ ∝ C^A^_k_/U^A^_k_

## Appendix 2

### Stata code for incorporating baseline survey characteristics

In Stata, the survey characteristics of the study must be declared prior to undertaking any analysis that acknowledges the sampling design. The command that brings the stratification, multistage design (at the PSU level) and sample weights together for the Ten to Men baseline data is:

where  is the variable representing the PSU (the SA1),  is the variable for the sample weight, and  is the stratification variable.

A population mean is then estimated with a command such as:

where  is a variable representing weight in kilograms (note that in the analyses reported here any values less than 0 kg or greater than 200 kg were set to missing prior to estimating the parameters). Similarly, a population proportion can be estimated with the command:

Measures of association (e.g. a regression coefficient, an odds ratio) is estimated with commands like:

where  represents the predictor self-rated health (in this case an indicator variable, hence the  prefix).
